# Application of the Population Pharmacokinetics Model-Based Approach to the Prediction of Drug–Drug Interaction between Rivaroxaban and Carbamazepine in Humans

**DOI:** 10.3390/ph16050684

**Published:** 2023-05-02

**Authors:** Lien Thi Ngo, Hwi-yeol Yun, Jung-woo Chae

**Affiliations:** 1 College of Pharmacy, Chungnam National University, Daejeon 34134, Republic of Korea; lienngovn@cnu.ac.kr; 2 Department of Bio-AI Convergence, Chungnam National University, Daejeon 34134, Republic of Korea

**Keywords:** rivaroxaban, carbamazepine, drug–drug interaction, population pharmacokinetics, allometric scaling

## Abstract

Rivaroxaban (RIV) is one of the direct oral anticoagulants used to prevent and treat venous and arterial thromboembolic events. Considering the therapeutic indications, RIV is likely to be concomitantly administered with various other drugs. Among these is carbamazepine (CBZ), one of the recommended first-line options to control seizures and epilepsy. RIV is a strong substrate of cytochrome P450 (CYP) enzymes and Pgp/BCRP efflux transporters. Meanwhile, CBZ is well known as a strong inducer of these enzymes and transporters. Therefore, drug–drug interaction (DDI) between CBZ and RIV is expected. This study aimed to predict the DDI profile of CBZ and RIV in humans by using a population pharmacokinetics (PK) model-based approach. We previously investigated the population PK parameters of RIV administered alone or with CBZ in rats. In this study, those parameters were extrapolated from rats to humans by using simple allometry and liver blood flow scaling, and then applied to back-simulate the PK profiles of RIV in humans (20 mg RIV per day) used alone or with CBZ (900 mg CBZ per day). Results showed that CBZ significantly reduced RIV exposure. The *AUC*_inf_ and *C*_max_ of RIV decreased by 52.3% and 41.0%, respectively, following the first RIV dose, and by 68.5% and 49.8% at the steady state. Therefore, the co-administration of CBZ and RIV warrants caution. Further studies investigating the extent of DDIs between these drugs should be conducted in humans to fully understand their safety and effects.

## 1. Introduction

Rivaroxaban (RIV; Xarelto, Janssen Pharmaceuticals, Beerse, Belgium) is categorized as a direct oral anticoagulant (DOAC) and is widely used for clinical purposes to prevent and treat venous and arterial thromboembolic events in several countries. It binds directly and reversibly to the active sites of both free and thrombin-bound forms of Factor Xa (FXa). Thereafter, it effectively blocks the activation of the coagulation cascade, preventing thrombus formation [1]. RIV is extensively metabolized by hepatic enzymes, and approximately 46% of the administered dose undergoes metabolic degradation. Cytochrome P450 (CYP) 3A accounts for approximately 18%, CYP2J2 for 14%, and CYP-independent hydrolysis (non–CYP-mediated hydrolysis of the amide bonds) for 14% of the administered RIV dose [2,3,4]. In addition, it is suggested that RIV is a substrate of P-glycoprotein (Pgp) and/or breast cancer resistance protein (BCRP) transporters [5,6,7,8]. An in vivo study by Gong et al. indicated that mice lacking expression of Pgp and BCRP transporters cleared RIV at a significantly reduced rate compared with normal mice. Moreover, a clinical study on metabolism and excretion of RIV in humans indicated that active renal transportation accounts for approximately 30% of the administered RIV dose [9]. With RIV being a potent substrate of both metabolic enzymes and transporters, the possibility of drug–drug interaction (DDI) is expected when RIV is co-administered with other drugs.

The DDI profiles of RIV concomitantly administered with ketoconazole (a strong inhibitor of both CYP3A4 and Pgp/BCRP) or ritonavir (a strong inhibitor of CYP3A4 and a weak inhibitor of Pgp/BCRP) have been reported [5], and an increase of up to 2.5-fold in the area under the plasma concentration–time curve (AUC) of RIV was observed compared with RIV administered alone. In addition, several case studies have reported adverse events when RIV was used in co-medication therapy [10,11,12,13]. For example, Becerra et al. [13] reported the first case of laboratory interaction between RIV and phenytoin, in which a patient suffered cerebral venous thrombosis after 1 week of concomitant use. The anti-FXa levels were considerably reduced to a lower-than-normal range. Based on the evidence from the literature and theoretical expectations of DDI potential, it is imperative to investigate the DDI profiles of RIV.

Over the last decades, pharmacokinetics (PK) model-based prediction using in vitro and in vivo data has increased significantly [14,15,16]. Two of the most widely used modeling methods are physiological-based pharmacokinetics (PBPK) modeling and population pharmacokinetics (PopPK) modeling. The PBPK model incorporates both the human physiological-related parameters and the drug-related information. Meanwhile, the PopPK model has the advantage of identifying the sources of variability in a drug kinetic profile, including those that are predictable (i.e., intrinsic factors (e.g., age, weight, and sex) and extrinsic factors (e.g., food and other drugs)) and unpredictable. Because of their advantages, the PBPK and PopPK models have simultaneously become two of the most widely used PK modeling methods.

Our group recently developed and published a PBPK model for RIV [17]. In that study, DDI profiles between RIV and carbamazepine (CBZ), one of the recommended first-line options for the treatment of seizures and epilepsy, were simulated in humans. CBZ is well known as a strong inducer of CYP enzymes [18,19] and Pgp transporters [20,21]. For example, CBZ interacts and significantly lowers the AUC of ivabradine (a strong CYP3A4 substrate) by about 80% [22] and fexofenadine (a strong Pgp substrate) by about 40% [23]. Results of our PBPK model-based approach showed that CBZ significantly reduces the exposure of RIV by 35.2% after the first RIV dose (AUC decreased from 2221.3 to 1438.7 ng · h/mL). In addition, our group recently conducted another study to investigate the DDI profile of RIV and CBZ in rats [24], indicating a decrease of 57.9% in RIV AUC due to pretreatment with CBZ. Using the PopPK model-based approach to predict the DDI profiles of RIV when used concomitantly with CBZ in humans was the aim of this study. Allometric scaling methods were applied to extrapolate the PK parameters of RIV in control and test groups (RIV administered in the absence/presence of CBZ, respectively) from rats to humans. Then, the corresponding PK profiles of RIV in humans were simulated with the extrapolated PK parameters using the PopPK model-based approach. The DDI results observed from the PopPK model in this study were then compared with those from the previous PBPK modeling to self-verify the accuracy of the two most widely used PK modeling methods.

## 2. Results

### 2.1. PopPK Model of RIV in Rats

**Figure 1:**
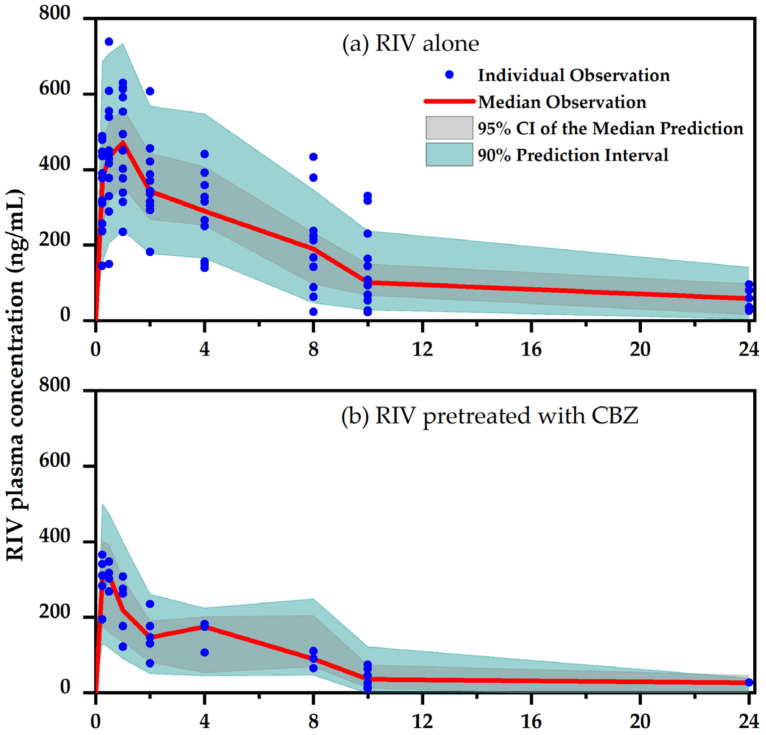
Pharmacokinetics of RIV in rats and visual prediction check for the developed PopPK model of RIV; (**a**) control group (RIV alone) and (**b**) test group (RIV administered with CBZ). This figure has been redrawn from our previous study result [24].

A study to investigate the effects of CBZ (and phenytoin) on PK of RIV in rats was reported previously. Within the scope of this study, we solely describe the parts related to the effects of CBZ. The PK study design and results are described in detail in our previous study [24]. Figure 1 shows the plasma concentration versus the time of RIV, demonstrating that CBZ significantly reduced the exposure of RIV. The mean *AUC* decreased from 3088.5 to 1299.3 ng · h/mL. The mean *C*_max_ decreased from 540.0 to 306.0 ng/mL.

**Figure 2:**
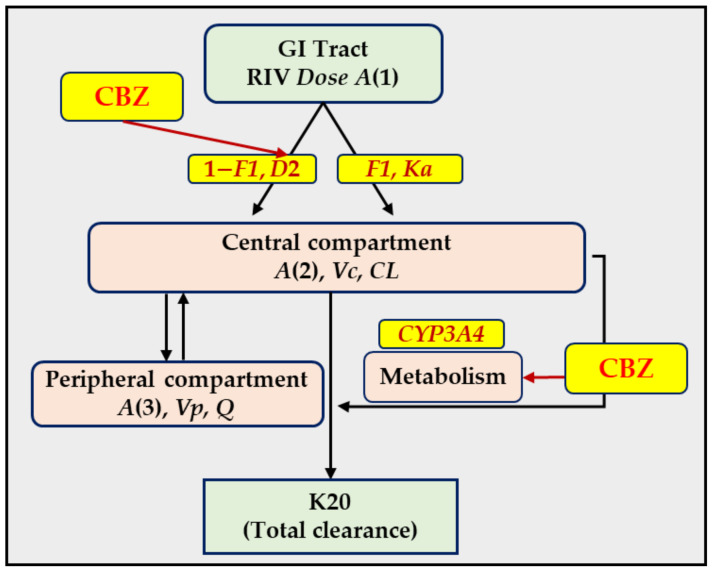
Schematic representation of the PopPK model for RIV and strategies to investigate the effect of CBZ on the PK of RIV. Notations are described in Table 1. This figure has been redrawn from the figure published in our previous study [24].

A PopPK model was developed to describe the PK of RIV in rats. The effect of pretreatment with CBZ as a covariate factor in the RIV PK was also investigated. Schematic representation of the PopPK model and the effect of CBZ on the PK of RIV are described in detail in our previous study [24] and illustrated in Figure 2. The final parameters estimates, their relative standard errors, and random errors are listed in Table 1.

In brief, the PK of RIV was described as a two-compartmental disposition model with a first-order elimination process. Mixed zero- and first-order kinetics were used for the absorption process. The zero-order kinetics (modeled by the absorption time *D2*) described the solubility limited absorption of RIV through the capillary network surrounding the enterocytes and hepatic portal vein. The first-order kinetics (modeled by the absorption rate constant *K*_a_) described the absorption of RIV (accompanying oil) entering the systemic circulation through the lymphatic system. The CBZ effects on the PK of RIV were modeled through the effects on the apparent clearance (increased *CL*/*F*) and the drug’s absorption (increased absorption time *D2*). The effects were determined using the following equations:

*CL*/*F* = 0.610 × (1 + *CL*_CBZ_) (1)

*D2* = 6.62 × (1 + *D2*_CBZ_) (2)

wherein *CL*_CBZ_ is 0 and 2.11 for the control and test groups, respectively, and *D2*_CBZ_ is 0 and 0.339 for the control and test groups, respectively. This means that CBZ increased the *CL*/*F* and *D2* of RIV by 211% and 33.9%, respectively, compared with RIV administered alone.

### 2.2. Extrapolation of RIV PK Parameters from Rats to Humans

The DDI profiles of RIV in humans were accessed by comparing the PK of RIV in the two groups: control (subjects received RIV alone) and test (subjects received RIV with CBZ) groups.

The PK parameters of clearance and volume of distribution of RIV in each group were extrapolated from rats to humans using allometric scaling methods. The first-order absorption rate constant was extracted from an RIV PK profile in humans [25]. Other parameters were assumed to be the same in rats and humans. The extrapolated parameters for RIV in humans are listed in Table 1. The volumes of distribution (*V*/*F*) of RIV in the central and peripheral compartments in both groups were 42.1 and 336 L, respectively. The apparent inter-compartment clearance in both groups was 9.86 L/h. The *CL*/*F* of RIV in the control and test groups were 9.03 and 28.1 L/h, respectively. The zero-order absorption rates were 6.62 and 8.84 h in the control and test groups, respectively.

### 2.3. Prediction of DDI Profile of RIV and CBZ in Humans

After all PK parameters in humans (Table 1) had been obtained, a simulation of 1000 replicates was performed to predict the PK profile of RIV following administration of 20 mg of RIV alone or with 900 mg/day of CBZ in humans by using the PopPK model-based approach. The variability of each PK parameter was assumed to be the same in humans and rats.

**Figure 3:**
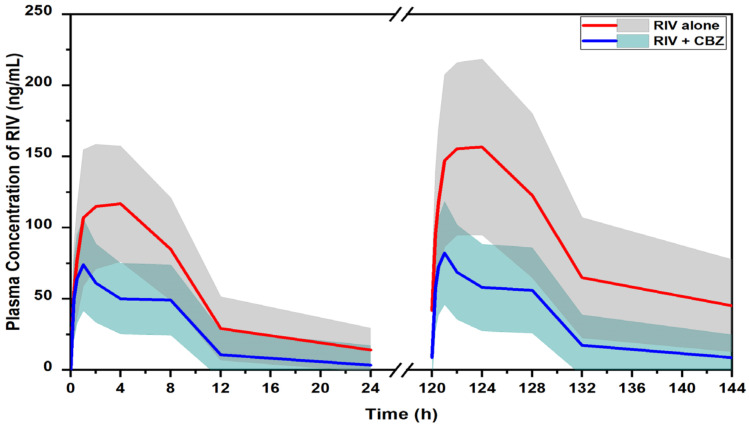
Predicted PK profiles of RIV in humans following oral administration of 20 mg/day of RIV in the first dose interval and the steady state with/without 900 mg/day of CBZ. Solid lines are mean values of the population. Shaded areas are 5th to 95th percentile values of the population.

The simulation results are presented in Figure 3 and Table 2, indicating CBZ significantly reduced the exposure of RIV when the two drugs were concomitantly used. When RIV was administered alone, the *AUCs* of RIV were 1291.7 and 2157.5 ng · h/mL in the first dosing interval and the steady state, respectively. In the presence of CBZ, the RIV *AUCs* were 615.7 ng · h/mL (decreased by 52.3%) and 775.2 ng · h/mL (decreased by 68.5%) in the first dosing interval and the steady state, respectively. Similarly, the *C*_max_ values of RIV were significantly decreased by 41% (from 133.2 to 78.6 ng/mL) after the first dose and by 49.8% (from 172.2 to 86.5 ng/mL) at the steady state. In addition, the results showed that in the presence of CBZ, RIV was eliminated from the body at a faster rate. The elimination half-life (*t_1_*_/*2*_) changed from 6.65 to 5.01 h.

$$\mathrm{Relative}\;\mathrm{change}=\left[1-\frac{PK\;Parameter_{\mathrm{RIV}\;\mathrm{with}\;\mathrm{CBZ}}\;}{PK\;Parameter_{\mathrm{RIV}\;\mathrm{alone}}}\right]\times 100\;\left(\%\right)$$

### 2.4. Comparison of PopPK and PBPK Model-Based Approaches

In a previous study, our team developed a PBPK model for RIV in humans. In that model, the metabolic degradation by CYP3A4, CYP2J2, and non-CYP enzymes, and the active transportation by Pgp/BCRP, were implemented [17]. To perform the simulation for the DDI profile of RIV and CBZ, the PBPK model for RIV was paired with a previously developed PBPK model for CBZ extracted from the OSP Library [26,27]. The simulation results from the PBPK model-based approach are listed in Table 2, indicating that CBZ significantly reduced the exposure of RIV. The *AUCs* of RIV were reduced by 35.2% (from 2221.3 to 1438.7 ng · h/mL) and by 25.5% (from 2467.3 to 1838.4 ng · h/mL) after the first dose and at the steady state, respectively. The *C*_max_ of RIV were reduced by 37.7% (from 266.3 to 166.1 ng/mL) and 36.4% (from 282.3 to 179.5 ng/mL), respectively.

In comparison with the results observed from the PopPK model-based approach, however, the decrease in RIV exposure predicted by the PBPK model was significantly less than the result predicted by the PopPK model-based approach; 35.2% versus 52.3% (after the first dose) and 25.5% versus 68.2% (at the steady state) were the decreases in the *AUCs* of RIV predicted by the PBPK versus PopPK model-based approach, respectively. Correspondingly, 37.7% versus 41.0% (after the first dose) and 36.4% versus 49.8% were the decreases in the *C*_max_ of RIV.

## 3. Discussion

Patients might experience unexpected side effects if DDI occurs because this phenomenon may make the drugs less effective (if exposure is reduced) or even harmful to health (if exposure is increased). Therefore, it is necessary to determine the DDI potential if co-medication therapy is applied. In the present study, we aimed to predict the DDI profiles of RIV when concomitantly used with CBZ by using the PopPK model-based approach. The RIV apparent clearances and volumes of distribution were extrapolated from rats to humans by using allometric scaling methods. PopPK modeling and simulation were then performed to simulate the RIV PK profiles in humans. Comparison of the RIV PK profiles when administered with/without CBZ enabled determination of the effects of CBZ on the RIV PK profile.

The administered doses in the PK study in rats were selected using the recommended doses for adults in clinical practice. The recommended dose of RIV for reducing the risk of stroke in nonvalvular atrial fibrillation is 20 mg once daily [28], and the recommended maintenance dosage of CBZ is 800–1200 mg daily for the indication of epilepsy [29]. Therefore, for DDI profiles, doses in humans were selected as follows: RIV at a dose of 20 mg/day (0.333 mg/kg, once daily) and CBZ at a dose of 900 mg/day (7.5 mg/kg, twice daily). Based on the body surface area of humans and rats (assuming body weights of 60 kg and 0.25 kg, respectively), a conversion factor of 0.164 was applied to calculate the equivalent doses for RIV and CBZ in rats [30], which were 2.0 mg/kg once daily for RIV and 45 mg/kg twice daily for CBZ. Considering the sensitivity of our analysis system, especially when CBZ was expected to reduce RIV exposure, the PK study in rats was conducted with an increased RIV dose of 3 mg/kg. The results of the study indicated that CBZ significantly reduced exposure to RIV. The mean *AUC* decreased by 57.9% (from 3088.5 to 1299.3 ng · h/mL), and the mean *C*_max_ decreased by 43.3% (from 540.0 to 306.0 ng/mL).

RIV is extensively metabolized by hepatic enzymes. Approximately 32% of the administered dose undergoes metabolic degradation by CYP enzymes (18% by CYP3A4 and 14% by CYP2J2) [2,3,4]. In addition, Pgp/BCRP transporters are involved in the active transportation of RIV. The efflux function of these transporters in the gastrointestinal tract, kidney, and liver significantly contributes to the PK of the drug. It is reported that active transportation accounts for about 30% of the RIV dose [9]. Meanwhile, CBZ is well known as a strong inducer of CYP enzymes [18,19] and also Pgp/BCRP transporters [20,21]. Therefore, CBZ is expected to increase the RIV elimination by increasing the metabolic degradation of CYP enzymes and the active renal elimination of RIV. CBZ is also expected to reduce the absorption rate of RIV in the gastrointestinal tract by increasing the transporters’ efflux function. Therefore, to model for the decrease in RIV exposure, pretreatment with CBZ as a covariate factor was tested for the *CL*/*F* (modeling for the elimination process) and *D2* and *K*_a_ (modeling for the absorption process). Results of the model developed confirmed that CBZ significantly increases the elimination (*CL*/*F* increased by 211%, from 0.609 to 1.894 L/h/kg) and decreases the absorption (prolonged the absorption time by 33.9%, from 6.62 to 8.84 h) of RIV.

PK parameters estimated in rats were extrapolated to humans by using allometric scaling methods. Because of its simplicity, simple allometric scaling has been intensively studied and widely applied to predict many important PK parameters (including *CL*, *V*, and *t*_1/2_). However, simple allometric scaling has limitations, especially since it assumes anatomical, physiological, and biochemical similarities among animals. In the case of drugs that exhibit species-specific differences, for instance, drugs with high protein binding properties, significant biliary excretion, extensive active renal secretion, active metabolism, or species-specific binding [31,32,33], the application of simple allometric scaling with a correction factor might be more suitable. RIV is one of these cases. The drug is primarily cleared by the hepatic metabolism [2,3,4]; therefore, an allometric scaling method with a correction factor for liver blood flow (LBF) to account for the difference in hepatic elimination functions between humans and rats was selected. This method assumes that drug clearance is proportional to the LBF of the subject. As a result, we used a simple allometric scaling method to extrapolate for *V* but an LBF allometric scaling method for *CL* in the case of RIV. In humans without the presence of CBZ, the extrapolated *CL*/*F* of RIV from the central compartment was 9.03 L/h. This is similar to that reported previously (9.2 L/h) [25]. The extrapolated *V*_c_/*F* of RIV in the central compartment was 42.1 L, also consistent with that in the literature (55.3 L) [25]. The first-order absorption rate constant was extracted from an RIV PK profile in humans [25]. Other parameters were assumed to be the same in rats and humans.

When all the PK parameters of RIV were extrapolated in humans, a simulation of 1000 replicates was performed to predict the PK profile of RIV in humans following administration of RIV at 20 mg/day with/without pretreatment with CBZ at 900 mg/day. The variability of each PK parameter was assumed to be the same in humans and rats. Following the first RIV dose, the *AUC* and *C*_max_ were predicted to be 1291.7 ng · h/mL and 133.2 ng/mL, respectively. These PK parameters were within two-fold of the clinically observed values extracted from the literature [5,34,35,36,37,38]. In detail, the observed *AUC* of RIV was 1847.5 ng · h/mL at the mean and ranged from 1559.6 to 2244.9 ng · h/mL. The observed *C*_max_ of RIV was 219.7 ng/mL at the mean, ranging from 189.9 to 291.6 ng/mL. At the steady state, the respective RIV PK parameters were predicted to be 2157.5 ng · h/mL and 172.2 ng/mL (Table 2). These values also lay within two-fold of the clinically observed values extracted from the literature [37,38]. In detail, the means (range) for the *AUC* and *C*_max_ of RIV were 2619.2 (2329.9–2908.4) ng · h/mL and 341.5 (273.5–409.5) ng/mL, respectively. These results indicated that the PopPK model and the extrapolation method could predict the PK profile of RIV in humans. Therefore, it would be applicable to investigate the DDI between RIV and CBZ in humans.

Results of the simulation for the effects of CBZ on the PK of RIV indicated that CBZ significantly reduced RIV exposure in humans. Respective decreases of 52.3% (from 1291.7 to 615.7 ng · h/mL) and 41.0% (from 133.2 to 78.6 ng/mL) in the *AUC*_inf_ and *C*_max_ were observed after the first RIV dose. At the steady state, the corresponding decreases were 68.5% (decreased from 2157.5 to 775.2 ng · h/mL) and 49.8% (decreased from 172.2 to 86.5 ng/mL).

DDI profiles of RIV and CBZ predicted from the PopPK model were then compared with those observed from the PBPK model-based approach [17]. The comparison showed agreement in predicting CBZ effects on the PK of RIV. Both the PopPK and the PBPK model-based approaches indicated that CBZ significantly reduced the exposure of RIV. However, the decrease in RIV exposure predicted by the PBPK model was significantly less than the result predicted by the PopPK model-based approach—35.2% versus 52.3% (after the first dose) and 25.5% versus 68.2% (at the steady state) for the *AUC* of RIV predicted by the PBPK versus the PopPK model-based approach, respectively. Correspondingly, 37.7% versus 41.0% (after the first dose) and 36.4% versus 49.8% (at the steady state) were the decreases in the *C*_max_ of RIV.

One reason for this phenomenon may be that the PBPK approach considered only the CYP3A4-mediated effect. Because of a lack of in vitro and in vivo experiments concerning the reaction, the Pgp transporter-mediated induction effect was not implemented in the developed CBZ model. As a result, the decreases in the PK parameters of RIV due to CBZ effects might have been under-predicted, while all possible (known and unknown) effects of CBZ were considered in the PopPK approach based on a real study conducted in rats [24]. This is a common limitation of the PBPK models. In the case of a DDI, it is not possible to predict the impact of concomitant drug administration if the enzymes involved in the metabolism of the individual drugs are unknown. In this study, it was well known that RIV is a substrate of Pgp/BCRP and CBZ an inducer of these transporters [20,21,39]. Further, the role of the Pgp induction effect in the DDI of RIV was supported by the results of the investigation conducted in rats [24]. In addition to the increased plasma clearance, CBZ also reduced the absorption rate of RIV by extension of the absorption time of the zero-order absorption process (from 6.62 to 8.86 h), which described the movement of the drug from the GI tract, crossing the basolateral membrane, and entering the systemic circulation via the capillary network surrounding the enterocytes and hepatic portal vein. The induced expression of the Pgp transporter in the intestinal tract affected the efflux of RIV, consequently limiting the rate and extent of drug absorption. This finding shows that in the case of RIV, because of its poor solubility, transporters play an important role in drug absorption.

The present study had certain limitations that need to be considered. Firstly, the induction effect of CBZ on CYP3A4 enzymes and Pgp transporters was solely tested in rats and then extrapolated to humans assuming that rats and humans share similar anatomical, physiological, and biochemical characteristics. However, it is important to note that animal populations may not precisely reflect human populations. Furthermore, RIV, the drug under investigation, is highly bound to plasma protein. The unbound fraction (fu) of RIV is 1.3% (the fraction binding is 98.7%) in rats and an average of 6.5% (the fraction binding is 92–95%) in humans [2]. Theoretically, only free drugs are available for metabolism (in hepatocytes) and elimination (in the kidney). Consequently, the disposition of RIV would be variable between rats and humans due to the interspecies differences in the fraction of drug unbound. However, to date, the conclusion regarding the consideration of fu as a correction factor in allometric scaling is still controversial [40,41,42]. In certain cases, it may be possible to predict unbound clearance (with the consideration of fu to correct the interspecies differences) more accurately than total clearance (without the fu consideration), or vice versa. In this study, the fu was not considered in allometric scaling for RIV because there was no specific information. Although interspecies allometric scaling has limitations and needs further refinements, the method is still a useful tool and rational option for the prediction of drug PK parameters and PK profiles in humans, particularly where there are no clinical data available. In this study, an evaluation step was performed to assess the extrapolation’s power in predicting RIV PK in humans, which showed reasonable agreement between the predicted and observed RIV PK in humans. As a result, the extrapolated PK results were applied to predict the DDI between RIV and CBZ. Based on the predicted DDI profiles, further clinical studies are required to fully comprehend the DDI profiles of rivaroxaban in patients to make decisions about dosage adjustments.

## 4. Materials and Methods

### 4.1. PK Study in Rats

The PK study design to investigate the effects of CBZ (and phenytoin) in rats is described in detail in our previous study [24]. Within the scope of this study, we describe solely the parts relating to the effects of CBZ. Rats were weighed and randomly divided into two groups (n = 6/group). Each rat received a single oral dose of RIV (3 mg/kg, once daily) without (control group) or with (test group) pretreatment with CBZ (45 mg/kg, twice daily) for 6 consecutive days. On Day 7, RIV was given 30 min after the morning dose of CBZ/placebo. Blood samples were collected before the RIV dose and then subsequently at 0.25 h, 0.5 h, 1 h, 2 h, 4 h, 8 h, 10 h, and 24 h after the RIV dose. For both groups, rats had access to water and food ad libitum. The animal study was approved by the Animal Ethics Committee of Chungnam National University (No. 2019012A-CNU-193, approved on 27 December 2019). All procedures were conducted in accordance with the assurance statement and guidelines in the National Institutes of Health’s Guide for the Care and Use of Laboratory Animals.

### 4.2. PopPK Model of RIV in Rats

A PopPK model was developed to describe the PK of RIV in rats. The effect of pretreatment with CBZ as a covariate factor in the RIV PK was also investigated. All the modeling and simulation were performed on NONMEM version 7.3.0 and executed through the PsN software tool (version 4.4.0) integrated into Pirana software [43].

The effects of CBZ on the PK of RIV were tested by introducing pretreatment with CBZ as a covariate in the elimination process (apparent clearance *CL*/*F*) and the absorption process (absorption time *D2* and/or absorption rate constant *K*_a_). The covariate was coded into the model as an index variable. For example, the categorical covariate was added to the model to determine the CBZ effect on RIV *CL*/*F*, as described in the following equation:

*CL* = *TVCL* × (1 + *CL*_CBZ_) (3)

wherein *TVCL* is the typical value in the subjects receiving RIV alone and *CL*_CBZ_ is the relative fractional change in *CL* accounting for the CBZ effects (*CL*_CBZ_ is 0 for the control group, and greater than 0 for the test group).

### 4.3. Extrapolation of RIV PK Parameters from Rats to Humans

The PK of RIV in rats were extrapolated to humans with the assumption that there are anatomical, physiological, and biochemical similarities among rats and humans. A simple inter-species allometric scaling method [44,45,46,47] and an LBF method [48] were applied to extrapolate the volumes of distribution ($V_{c}$ and $V_{p}$) and clearances (inter-compartmental *Q* and systemic clearances *CL*) of RIV, respectively, to humans. In detail, the volume of distribution of RIV in humans was defined by the following equation:

(4) $$V_{\mathrm{human}}=V_{\mathrm{rat}}\times {\left(\frac{BW_{\mathrm{human}}}{BW_{\mathrm{rat}}}\right)}^{1.00}$$

wherein *BW*_human_ and *BW*_rat_ are body weight of the human and the rat, respectively, and average values of 60 kg and 0.25 kg were assumed. Clearances were considered proportional to LBF and extrapolated following a method described by Ward and Smith [48], as described by the following equation:

(5) $$CL_{\mathrm{human}}=CL_{\mathrm{rat}}\times \left(\frac{LBF_{\mathrm{human}}}{LBF_{\mathrm{rat}}}\right)$$

wherein *LBF* values of rats and humans are 85 and 21 mL/min/kg of body weight, respectively [48]. The effects of CBZ on the PK of RIV in humans were assumed to be the same as in rats. The rate constant of the first-order absorption process was acquired from a published study [25]. After obtaining all PK parameters of RIV in humans, a simulation of 1000 replicates was performed to predict the PK profile of RIV following administration of RIV 20 mg alone or with CBZ 900 mg/day in humans by using the PopPK model-based approach.

### 4.4. Evaluation of the Extrapolation Results in Humans

To determine whether the extrapolation from rats to humans was successful or not, the extrapolated PK parameters were compared with observed clinical values in humans. The extrapolation was considered successful when the extrapolated human PK parameters (*AUC*_inf_ and *C*_max_) were within two-fold of the experimental values. Otherwise, it was assessed to be unsuccessful [49,50,51,52]. In the present study, clinical datasets (those used for the development and evaluation of the PBPK model) following a single oral dose of 20 mg RIV under the fed state were collected to evaluate the extrapolation from rats to humans.

## 5. Conclusions

In conclusion, CBZ was predicted to significantly decrease the exposure of RIV when concomitantly administered with RIV in humans (CBZ 900 mg/day, and RIV 20 mg/day). Respective decreases after the first dose and at the steady state of 52.3% (from 1291.7 to 615.7 ng · h/mL) and 68.5% (from 2157.5 to 775.2 ng · h/mL) in *AUC*_inf_, and 41.0% (from 133.2 to 78.6 ng/mL) and 49.8% (from 172.2 to 86.5 ng/mL) in *C*_max_, were predicted. These results agree with the results obtained by applying the PBPK model-based approach. Studies on DDI between RIV and CBZ should be conducted in humans to obtain a full understanding of their safety and effects.

## Figures and Tables

**Table 1:** PK parameters of RIV extrapolated from rats to humans.

Description	Estimated in Rats [24]	Extrapolated in Humans	RSE (%)
PK parameters			
*CL*/*F* in control group	0.609 (L/h/kg)	9.03 (L/h)	36.9
*CL*/*F* in test group	1.894 (L/h/kg)	28.1 (L/h)	69.7
$V_{c}$/*F*	0.701 (L/kg)	42.06 (L)	44.2
*Q*/*F*	0.665 (L/h/kg)	9.86 (L/h)	21.4
$V_{P}$/*F*	5.60 (L/kg)	336 (L)	38.1
*D2* in control group	6.62 (h)	Same	
*D2* in test group	8.84 (h)	Same	17.2
*K*_a_	2.31 (1/h)	0.97 [25]	33.6
*F1*	0.260	Same	9.10
*Alag2*	0.501 (h)	Same	
Inter-individual variability (IIV)			
$\mathrm{IIV}\;\mathrm{for}\;V_{c}$/*F*	47.0 (%)	Same	33.0
IIV for *CL*/*F*	49.0 (%)	Same	35.0
Residual error			
Additive error	13.6 (ng/mL)	Same	35.8
Proportional error	23.2 (%)	Same	17.2

*CL*/*F*, apparent clearance from central compartment; *V*_c_/*F*, volume of distribution of RIV in central compartment; *Q*/*F*, apparent clearance between central and peripheral compartment; *V*_p_/*F*, volume of distribution of RIV in peripheral compartment; *D2*, time of the zero-order absorption; *K*_a_, rate constant of the first-order absorption; RSE, relative standard error; *F1*, fraction of RIV absorbed following the first-order kinetics; *Alag2*, delay time of the zero-order absorption; *F*, bioavailability of RIV. PK parameters in rats were adopted from our previous study [24]. *K_a_* was obtained from a study by Mueck et al. [25].

**Table 2:** Predicted PK parameters of RIV following oral administration of 20 mg/day of RIV in the first dose interval and the steady state with/without 900 mg/day of CBZ.

Parameters		Unit	RIV alone	RIV + CBZ	Relative Change (%)
PopPK model-based approach	*AUC* *___*_first_	ng · h/mL	1291.7 (770.9–1837.0)	615.7 (296.8–1025.4)	52.3
	*AUC* *___*_SS_	ng · h/mL	2157.5 (983.6–3756.9)	775.2 (323.8–1463.1)	68.5
	*C*_max_first_	ng/mL	133.2 (88.1–185.4)	78.6 (45.8–121.0)	41.0
	*C*_max_SS_	ng/mL	172.2 (101.5–259.9)	86.5 (48.3–136.1)	49.8
	*t_1_*_/*2*_	h	6.65	5.01	-
PBPK model-based approach *^¥^*	*AUC___*_first_	ng · h/mL	2221.3 (488.3–4087.9	1438.7 (338.8–3002.0)	35.2
	*AUC___*_SS_	ng · h/mL	2467.3 (544.6–4667.7)	1838.4 (463.8–3684.4)	25.5
	*C*_max_first_	ng/mL	266.3 (80.7–452.3)	166.1 (54.1–299.4)	37.7
	*C*_max_SS_	ng/mL	282.3 (86.2–473.6)	179.5 (59.0–321.5)	36.4
	*t_1_*_/*2*_	h	12.9	15.5	-

Data are presented as mean (5th percentile–95th percentile), except *t*_1/2_. *^¥^* Results from the PBPK model-based approach were extracted from our previous study [17]. The subscriptions “first” and “SS” stand for “first dose” and “steady state”, respectively; *AUC*, area under the concentration–time curve calculated during one dosing interval; *C*_max_, maximum concentration; *t_1_*_/*2*_, elimination half-life.

## Data Availability

The data presented in this study are openly available in MDPI at [https://doi.org/10.3390/pharmaceutics12111040], reference number 24.
